# Implementation of seven echocardiographic parameters of myocardial asynchrony to improve the long-term response rate of cardiac resynchronization therapy (CRT)

**DOI:** 10.1186/1476-7120-6-58

**Published:** 2008-11-25

**Authors:** Fabian Knebel, Sebastian Schattke, Hansjürgen Bondke, Christoph Richter, Christoph Melzer, Henryk Dreger, Andrea Grohmann, Gert Baumann, Adrian C Borges

**Affiliations:** 1 Medizinische Klinik für Kardiologie und Angiologie, Campus Mitte, Charité – Universitätsmedizin Berlin, Germany

## Abstract

**Background:**

Cardiac resynchronization Therapy (CRT) is an effective therapy for chronic heart failure with beneficial hemodynamic effects leading to a reduction of morbidity and mortality. The responder rates, however, are low. There are various and contentious echocardiographic parameters of myocardial asynchrony. Patient selection by echocardiographic assessment of asynchrony is thought to improve responder rates.

**Methods:**

In this small single-center pilot-study, seven established parameters of myocardial asynchrony were used to select patients for CRT: (1) interventricular electromechanical delay (IMD, cut-off ≥ 40 ms), (2) Septal-to-posterior wall motion delay (SPWMD, ≥ 130 ms), (3) maximal difference in time-to-peak velocities between any two of twelve LV segments (Ts-12 ≥ 104 ms), (4) standard deviation of time to peak myocardial velocities (Ts-12-SD, ≥ 34.4 ms), (5) difference between the septal and basal time-to-peak velocity (TDId, ≥ 60 ms), (6) left ventricular electromechanical delay (LVEMD, > 140 ms) and (7) delayed longitudinal contraction (DLC, > 2 segments).

16 chronic heart failure patients (NYHA III–IV, LVEF < 0.35, QRS ≥ 120 ms) at least two out of seven parameters of myocardial asynchrony received cardiac resynchronization therapy (CRT-ICD). Follow-up echo examination was after 6 months. The control group was a historic group of CRT patients (n = 38) who had not been screened for echocardiographic signs of myocardial asynchrony prior to device implantation.

**Results:**

Based on reverse remodeling (relative reduction of LVESV > 15%, relative increase of LVEF > 25%), the responder rate to CRT was 81.2% in patients selected for CRT according to our protocol as compared to 47.4% in the control group (p = 0.04). At baseline, there were on average 4.1 ± 1.6 positive parameters of asynchrony (follow-up: 3.7 [± 1.6] parameters positive, p = 0.52). Only the LVEMD decreased significantly after CRT (p = 0.027). The remaining parameters showed a non-significant trend towards reduction of myocardial asynchrony.

**Conclusion:**

The implementation of different markers of asynchrony in the selection process for CRT improves the hemodynamic response rate to CRT.

## Background

Cardiac resynchronization Therapy (CRT) is an effective therapy for chronic heart failure with beneficial hemodynamic effects and a reduction of morbidity and mortality [1-7]. Despite the overall favorable effect of CRT and a lack of a clear definition of hemodynamic benefit, published responder rates in the large CRT studies are low (43–69%) [8-10]. In the CARE-HF study, only about 50% of the patients responded clinically. The COMPANION study [1] did not publish responder rates (2004).

In the CARE-HF study [6] with an observation period of 18 months, about half of the CRT patients had worsening of heart failure, 21% died (of any cause), and 13% had complications caused by CRT (e.g., lead displacement, pneumothorax, infection, dissection, and one implantation-related death). Furthermore, as CRT is an expensive therapy with relatively high complication rates, careful patient selection and definition of predictors are needed.

Accounting for the complexity of the parameters of asynchrony and the lack of a clear predictor, the indications for CRT in the current guidelines do not include echo parameters of myocardial asynchrony [11] and an expert statement of the ASE justifies patient selection by echo only in borderline cases [12].

There is no diagnostic gold standard to determine myocardial asynchrony. Echocardiography is considered as an objective tool to evaluate myocardial intra- and interventricular asynchrony. However, when assessing myocardial asynchrony, some parameters cannot be reliably measured in all patients. Integrating multiple parameters might therefore improve the diagnostic sensitivity and specificity of asynchrony tests in the individual patient.

The goal of this pilot study was to improve the response rate to CRT by prospective patient selection by echocardiography.

## Methods

This is a prospective single center study evaluating the improvement of CRT response by implementing a new echocardiographic approach in patient selection for CRT-ICD.

### Patients

Inclusion criteria were: chronic heart failure in the functional classes NYHA III-IV with a LVEF < 0.35, QRS > 120 ms and at least two positive echo parameters of myocardial asynchrony (combination of two intraventricular or combination of one inter- and one intraventricular parameter). Patients with more than two myocardial segments of akinesia (suggesting scar tissue) or left ventricular aneurysms (end-diastolic thinning of the myocardium < 5 mm) were excluded from this study.

In order to obtain unbiased data regarding cardiac improvement, standard and individually optimized heart failure medication (including beta blockers, ACE inhibitors or AT-1 receptor blockers at the maximally tolerated dose and spironolactone, 25 mg/d) remained unchanged 3 months prior to implantation of CRT in all patients. Follow-up was performed after 6 months.

The historic control group (n = 38) comprised patients from our hospital that had received CRT in recent years. The inclusion criteria for these patients were identical: NYHA III-IV with a LVEF < 0.35 and a QRS width > 120 ms. All patients received a CRT- ICD and were fully documented by 2D and Tissue Doppler echocardiography at baseline. The CRT patients underwent 6 months follow-up examination, including echocardiography.

The AV-interval was set individually in all patients according to [13]. There was no V-V-delay in all patients.

### Echocardiography

Echocardiography was performed on a Vivid 7 (GE Vingmed, Horton, Norway). The images were stored digitally and analyzed off-line by EchoPac PC Dimension (GE Vingmed, Horton Norway). For TDI and 2D echocardiography analysis, three beats were stored.

Seven parameters of intra- and interventricular asynchrony were used [see table 1]. The parameters were chosen according to their previous evaluation in larger CRT studies. Furthermore, these parameters should be reproducible and technically easy to measure. If more than two of the seven parameters were positive, the patients received CRT.

**Table 1:** Echocardiographic parameters of myocardial asynchrony used to determine the presence of relevant asynchrony.

**Parameter**	**Definition**	**Cut-off**	**Ref**.	**Example**
Interventricular electromechanical delay (IMD)	Interventricular electromechanical delay between pulmonary and aortic outflow. Measured as the time between the onset of the QRS to onset of aortic flow vs. QRS to onset of pulmonary flow	≥ 40 ms	**6**	**1**

Septal-to-posterior wall motion delay (SPWMD)	M-mode: the interval between the maximum contraction of the septum and the maximum contraction of the left ventricular posterior wall	≥ 130 ms	**14, 15**	**2**

Ts-12	Maximum difference in Ts between any 2 of 12 LV segments	≥ 104 ms	**16**	**3**

Ts-12-SD	Standard deviation of the time to peak myocardial velocity (Ts) of the 12 LV segments	≥ 34.4 ms	**16**	**4**

Septal-to-lateral delay in peak systolic velocity (TDId)	Delay between the basal septal and basal lateral Ts, assessed with color-coded TDI	≥ 60 ms	**17**	**5**

LVEMD	aortic pre-ejection delay measured as the time between the onset of the QRS to beginning aortic flow	> 140 ms	**6**	**6**

Delayed longitudinal contraction (DLC)	Number of LV segments with DLC assessed by tissue tracking in the apical 4-chamber, 2-chamber and long-axis view	≥ 2	**18**	**7**

The interventricular electromechanical delay (IMD) is the time difference between the left ventricular and right ventricular electromechanical delays (i.e., the interval from the beginning of the QRS complex to the beginning of ejection in the pulmonary/aortic ventricular outflow tract). The cut-off value for interventricular asynchrony is > 40 ms [[6], additional file 1].

The septal-to posterior wall motion delay (SPWMD) is obtained from M-mode in the parasternal long axis as the shortest interval between the maximal contraction of the septum and the maximal contraction of the left posterior wall. The cut-off value for interventricular asynchrony is ≥ 130 ms [[14,15], additional file 2].

The maximal difference in time-to-peak velocities between any two of twelve LV segments (Ts-12) and the standard deviation of time-to-peak myocardial velocity (Ts-12-SD) are assessed with Tissue Synchronization Imaging (TSI). 12 myocardial segments in the apical views (apical 4-chamber, 2-chamber and long-axis view) are analyzed. These parameters with a cut-off value ≥ 34.4 ms (Ts-12-SD) and ≥ 104 ms (Ts-12) were previously prescribed as powerful predictors of response to CRT [[16], additional file 3,4]

The difference between the basal septal and basal lateral Ts is assessed with TDI in the apical four chamber view by color-coded TDI (TDId). The cut-off value ≥ 60 ms is a predictor of successful CRT [[17], additional file 5].

The left ventricular electromechanical delay (LVEMD) or aortic pre-ejection delay is measured as the time between the onsets of the QRS to aortic flow. The cut-off value of this parameter is > 140 ms indicating intraventricular asynchrony [[6], additional file 6].

The delayed longitudinal contraction (DLC) is assessed with tissue tracking in the apical four chamber, two chamber and long-axis views for the detection of LV segments with mechanical asynchrony. The highest possible frame rate was chosen according to [[17], additional file 7]. We considered two and more segments with DLC as indicative of asynchrony.

According to previous studies, we defined successful resynchronization therapy as a relative reduction of the LV end-systolic volume of more than 15% and a relative increase of the LVEF of more than 25% compared to baseline [19-21]. The latter has been evaluated in a follow-up interval of 6 months. The LVEF and left-ventricular volumes were calculated according to Simpson's rule [22].

Written consent was obtained from each patient, and the ethics committee of the Charité University Hospital approved the protocol.

### Statistics

Statistics were calculated using SPSS (version 12.0, Chicago, Ill, USA). Results are expressed as mean (± standard deviation). Comparisons of non-parametric variables between the "echo-guided CRT" patients and the control group were calculated by Wilcoxon test. The comparison of echocardiographic parameters between groups was calculated by unpaired Whitney-Mann test. Dichotomized data were analyzed by the Chi^2^-test. The level of significance was p ≤ 0.05.

## Results

16 patients with more than two positive echocardiographic parameters of asynchrony received an ICD-CRT ("echo-guided CRT" group).

The historic controls (n = 38) were chosen because of the complete echocardiographic follow-up at 6 months after CRT. The controls did not differ in regard to their baseline data: age, baseline QRS width and LVEF. However, there were more female patients in the control group (p = 0.04). (Table 2)

**Table 2:** Baseline patient characteristics (Differences between groups by Whitney-Mann test, Dichotomized data were analyzed by the Chi^2^-Pearson-Test)

	**echo-guided CRT group**	**controls**	**p**
n =	16	38	

% ischemic cardiomyopathy (ICMP)	37.5 (n = 5)	21.1 (n = 8)	0.49

Age [years]	67.1 (± 9.9)	63.6 (± 10.8)	0.79

male %	93.8 (n = 15)	68.4 (n = 26)	0.04

QRS width [mm] at baseline	166 (± 13)	165 (± 18)	0.48

Follow-up-interval [months]	6.7 (± 7.2)	9.4 (± 3.0)	< 0.001

LVEF [%]	23.1 (± 6.7)	24.5 (± 7.4)	0.75

LV-EDV [ml]	259.1 (± 111.2)	262.0 (± 104.8)	0.98

LV-ESV [ml]	199.04 (± 89.4)	204.3 (± 90.8)	0.38

% responder according to reduction of LVESV/increase in EF	13/16 = 81.2%	18/38 = 47.4%	0.04

**Figure 1:**
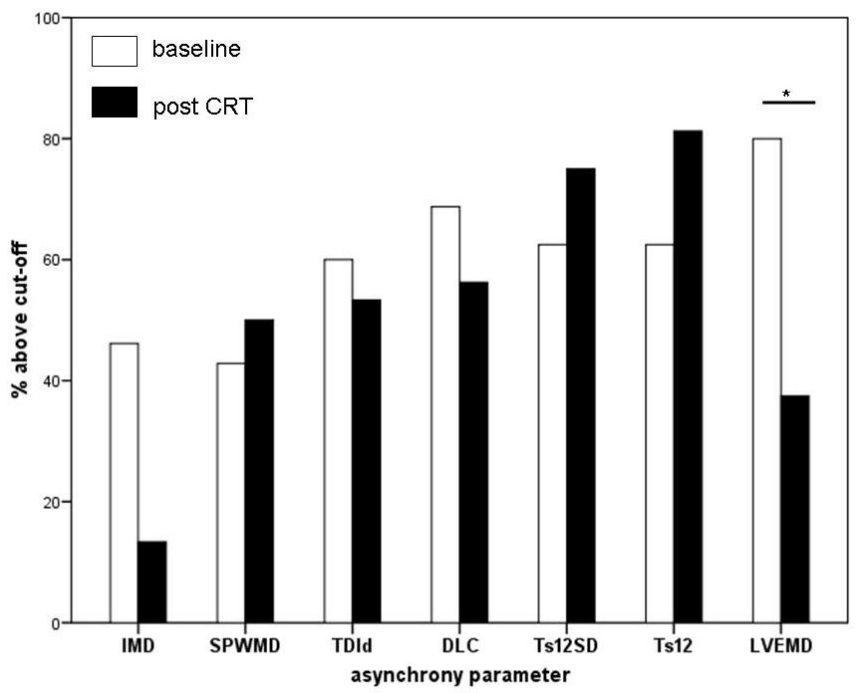
Percentage of positive findings of asynchrony parameters at baseline (white bars) and after 6 months of CRT (black bars). The cut-offs for each parameter are listed in table 1.

**Figure 2:**
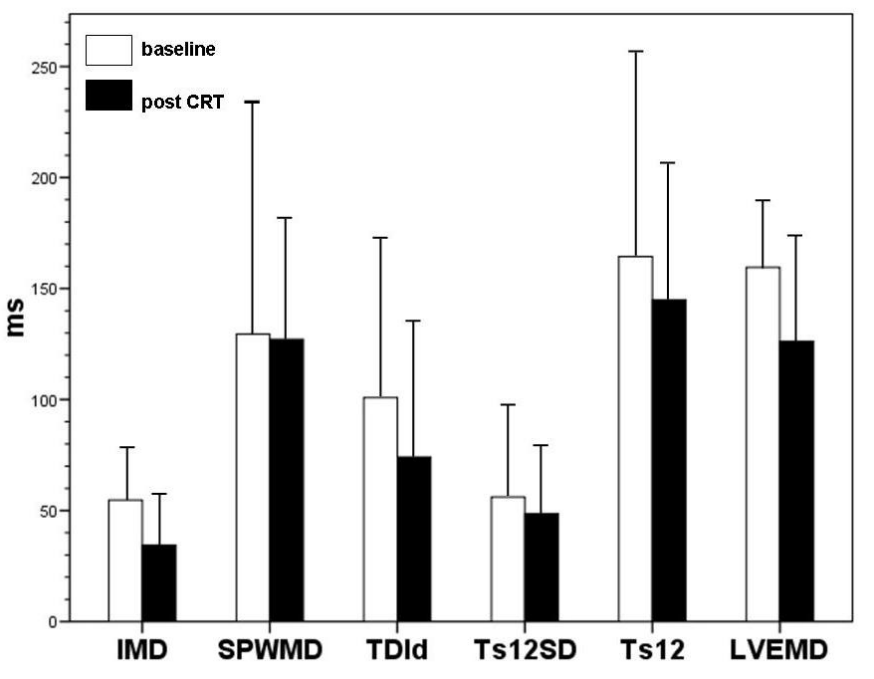
Times and delays in milliseconds at baseline (white bars) and after CRT (black bars). Mean ± SD. Data for DLC not shown (see table 4).

The echo-guided CRT-group had 4.1 ± 1.6 positive parameters of asynchrony at baseline (Table 3, figure 1, 2). After 6 months of CRT, 3.7 (± 1.6) parameters were positive (p = 0.52). Only the LVEMD decreased significantly after CRT (p = 0.027). The remaining parameters showed a non-significant trend towards reduction of myocardial asynchrony.

**Table 3:** Hemodynamic changes after CRT in the "echo-guided CRT group" and the control group.

	echo-guided CRT group			Controls			
	*baseline*	*Post CRT*	*p*	*baseline*	*Post CRT*	*p*	*P (control vs. new post)*

LVEF [%]	23.1 (± 6.7)	36.9 (± 11.2)	0.002	24.5 (± 7.4)	30.2 (± 10.2)	0.002	0.04

LV-ESV [ml]	204.3 (± 90.8)	153.8 (± 89.6)	0.023	262.0 (± 104.8)	199.0 (± 89.4)	0.030	0.378

LV-EDV [ml]	262.0 (± 104.8)	232.5 (± 104.5)	0.352	204.3 (± 90.8)	247.6 (± 119.7)	0.187	0.574

Amount of positive echo parameters of asynchrony	4.1 (± 1.6)	3.7 (± 1.6)	0.525	n.a.	n.a.		

**Figure 3:**
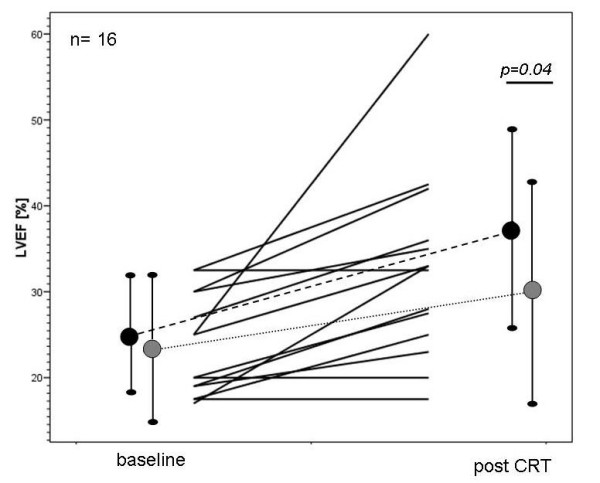
Echocardiographic improvement after CRT: Individual LVEF at baseline and after CRT in the in the echo-guided CRT group (n = 16, solid lines). The mean LVEF (± SD) in the echo-guided CRT group is shown in black (dashes line) and for the controls in grey (dotted line).

There was a clinical improvement in the echo-guided CRT group (baseline NYHA 3.1 ± 0.25, NYHA 1.8 [± 1.0] after CRT, p = 0.02). The responder rate in the "echo-guided CRT" group was 81.2% compared to 47.4% in the control group (p = 0.04). The reverse remodeling after CRT was significant in both groups (figure 3). Mitral regurgitation decreased in the echo-guided CRT group (2.2 ± 0.8 to 1.8 ± 0.023 after CRT, p = 0.023), whereas there was no significant change in the control group.

ROC analysis for the 7 parameters (value above cut-off measurements for asynchrony) to predict CRT benefit is presented as area-under-the-curve: IMD: AUC = 0.58, p = 0.612; SPWMD: AUC 0.46, p = 0.71; TDId: AUC 0.63, p = 0.79; DLC AUC 0.60, p = 0.93; Ts-12 AUC = 0.88, p = 0.25; LVEMD AUC 0.56, p = 0.33, TS-12-SD AUC = 0.88 p = 0.01.

Intra- and interobserver variability was calculated by the intra-class correlation coefficient (ICC). There were good correlations for Tissue Doppler measurements (ICC = 0.94, p < 0.001 and 0.96, p < 0.001, respectively).

## Discussion

The main result of this prospective pilot study is that prospective assessment of multiple echocardiographic parameters of asynchrony increases the success rate of CRT.

In most large studies, patient selection was solely based on QRS width and NYHA class. Patient selection by echocardiographic parameters of asynchrony was only performed in a subset of studies for borderline cases [6,8]. The predictors of successful CRT are heterogeneous and include a variety of clinical and echocardiographic parameters.

How can the improved responder rate in our study be explained? Firstly, as myocardial asynchrony is a complex phenomenon, every single parameter reflects one aspect of this pathology. Our parameters evaluate inter- and intraventricular asynchrony, 2D as well as TDI-echocardiography. Therefore, integrating different parameters in the selection process increases the chance to select patients with relevant asynchrony for CRT.

Secondly, the improved response rate could be due to the single center design and the careful patient assessment and low inter- and intra-observer-variability. TDI measurements are susceptible to observer variability, subjective assessment of the curves and individual learning curves. This might limit the reliability of multi-center studies in CRT as in the PROSPECT study. Different echo machines and unclear definition of echo parameters might also have contributed to disappointing results.

Thirdly, to our experience, it is difficult to rely on one parameter alone because, for example, the SPWD cannot be measured in patients with previous anterior and septal myocardial infarction. Furthermore, reduced acoustic windows limit certain measurements frequently.

In our study, most parameters showed a non-significant trend of improvement. This could be due to the small patient number in our study. But this might also indicate that a single parameter does not have enough power to predict response to CRT.

Two large studies have analyzed echocardiography in the prediction of CRT success: The PROSPECT and Re-THIN-Q study [8,9]. The parameters used in the PROSPECT study included SPWMD, IMD, left ventricular filling time in  relation to cardiac cycle length, LVEMD, TDId, and Ts-SD. These were comparable to the parameters used in this study. The negative result of the PROSPECT study (no single echo parameter can predict response to CRT) might have been caused by a high inter-core-lab variability (range 6.5–72.1%) that potentially blurred the role of echocardiography.

The success rate of CRT is low (50.3–63.0%) when defined by LVESD reduction [9]. The PROSPECT study design did not prospectively select patients according to presence of mechanical asynchrony but compared different echo parameters of asynchrony in their predictive value. In contrast, in our study, only patients with at least two positive findings of myocardial asynchrony were included to receive CRT.

The Re-THIN-Q study [8] evaluated CRT in patients with thin QRS complexes and evidence of mechanical asynchrony in Tissue Doppler imaging. The primary endpoint was increase in peak oxygen consumption of at least 1.0 ml/kg KG/min after 6 months. The response rate to CRT – defined as an increase in peak oxygen consumption – was 43%. In Re-THIN-Q, only two echo parameters were assessed to measure asynchrony (TDI derived mechanical delay in the septal-to-lateral and anteroseptal-to-posterior walls and the septal-to-posterior wall delay in M-mode). The TDI derived criterion was positive in 96% of patients, the M-mode derived criterion only in 4% of patients. The primary endpoint of the study was only met by the patients with QRS ≥ 120 ms. The echocardiographic parameters did not differ significantly in the responder and non-responder groups indicating that echo did not have a predictive value to select patients for CRT.

Which parameters contribute to patient selection for CRT? The optimal technology to measure myocardial asynchrony is still in debate. There is increasing concern about asynchrony measurement by TDI-velocity because of double-peaks and beat-to-beat variability of the curves which are subject to tethering and translational motion. Hence, some authors have suggested that strain and 2D strain derived measurements of asynchrony (which reflect local contractility) might have a higher prognostic value [23-25]. A recent study has shown an overlap of TDI-derived asynchrony parameters used in the large CRT studies in healthy controls and heart failure patients with LBBB [25]. This underlines that the current cut-offs need verification. Our study did not use 2D strain parameters for the detection of asynchrony but focused on parameters that were established in large clinical trials.

The study by Bleeker [26] suggested that myocardial scar burden and transmurality of the scar predicts CRT outcome. In our study, we did not systematically screen the patients by MRT prior to CRT implantation. However, we excluded patients with akinesia in more than two segments, myocardial aneurysms or left ventricular thinning in baseline echocardiography.

Suboptimal lead position can contribute to non-successful CRT. Studies have shown that optimal lead position in the site of the latest myocardial activation can improve the echocardiographic and clinical results [27,28]. In our study, the LV-lead position was not individually positioned. We aimed to implant the LV-lead in the postero-lateral coronary sinus vein in all patients.

## Conclusion

In summary, we conclude that the implementation of different markers of asynchrony seems to improve the hemodynamic response rate in this small single center study. To further support our findings, larger prospective, controlled and randomized multi-center studies will have to be performed in order to find an appropriate combination of parameters.

## Limitations

This is a small pilot study performed in a single center. Due to the small number of patients, no multivariate analysis of the several parameters and no calculation of predictive values could be performed. We have not systematically examined myocardial scar burden by cardiac MRI prior to CRT implantation. The choice of echo parameters should include parameters of three-dimensional echo as well as parameters of radial and circumferential asynchrony. This was not done because the software and equipment was not available for all patients.

## Abbreviations

CRT: cardiac resynchronization therapy; DLC: delayed longitudinal contraction; ICD: implantable cardioverter defibrillator ; IMD: interventricular electromechanical delay; LVEMD: left ventricular mechanical delay; ROC: Receiver Operator Characteristic; SPWMD: septal-to-posterior wall motion delay; TDI: tissue Doppler imaging; TDId: delay of the septal and basal time-to-peak velocity; Ts: time-to-peak systolic velocity; Ts-12: maximal difference in the time-to-peak velocities of 12 myocardial segments; Ts-12-SD: standard deviation of time-to-peak velocities of 12 myocardial segments; TSI: Tissue Synchronization Imaging;

## Authors' contributions

FK and SS equally contributed to the study. FK, SS, ACB, AG, CR, HJB, CM, HD, AP participated in contributions to conception, analysis and interpretation of data, the follow-up echocardiographic examinations and made comments to the manuscript. HB and CM implanted the CRT systems and analyzed data. BG has supervised and commented the study. ACB was the supervisor of echo examinations, is head of the echo lab, and contributed by revising the manuscript critically.

## Competing interests

The authors declare that they have no competing interests.

**Table 4:** Change of asynchrony parameters (pre-post) in the echo-guided CRT group, p (Wilcoxon test)

Parameter	baseline	post CRT	p
IMD [ms]	54.7 (± 26.8)	34.4 (± 23.8)	0.091

SPWMD [ms]	129.5 (± 106.3)	127.1 (± 53.2)	1.00

Ts-SD [ms]	56.1 (± 40.5)	48.5 (± 30.0)	0.691

Ts-12 [ms]	164.4 (± 91.8)	144.9 (± 66.1)	0.569

TDId [ms]	100.9 (± 71.4)	74.1 (± 61.5)	0.235

LVEMD [ms]	159.5 (± 30.7)	126.2 (± 49.0)	**0.027**

DLC [n]	1.7 (± 1.0)	1.3 (± 1.0)	0.394

**Table 5:** Accessibility of the asynchrony parameters at baseline and after CRT. Chi^2 ^test (p)

Parameter	baseline	Positive (%)	post CRT	Positive (%)	p
IMD [ms]	13	6/46.2%	15	2/13.3%	0.773

SPWMD [ms]	13	6/46.2%	14	7/50.0%	0.559

Ts-SD [ms]	16	10/62.5%	16	12/75.0%	0.119

Ts-12 [ms]	16	10/62.5%	16	13/81.3%	0.838

TDId [ms]	15	9/60.0%	15	8/53.3%	0.551

LVEMD [ms]	15	12/80.0%	16	6/37.5%	0.869

DLC [n]	16	11/68.8%	15	9/60.0%	0.171

## Supplementary Material

Additional File 1 Measurement of the IMD in the aortic and the pulmonary outflow tracts. Click here for file

Additional File 2 M-mode in the paarasternal long axis to determine SPWMD. Click here for file

Additional File 3 Assessment of Ts12 and Ts-12-SD by TSI. Click here for file

Additional File 4 TSI: Patient with asynchrony in the lateral wall (apical four chamber view). Click here for file

Additional File 5 TDId in a healthy control and the asynchrony of myocardial contraction in the septal and lateral basal segments. Click here for file

Additional File 6 Measurement of the LVEMD in the aortic outflow tract. Click here for file

Additional File 7 Apical four chamber view. Myocardial systolic displacement: Evidence for lateral apical wall delayed longitudinal contraction. Click here for file
