# Supplementary figures and images for: Implementation of seven echocardiographic parameters of myocardial asynchrony to improve the long-term response rate of cardiac resynchronization therapy (CRT)
Source: Cardiovasc Ultrasound. 2008 Nov 25;6:58. doi: 10.1186/1476-7120-6-58 (PMC2613384; doi:10.1186/1476-7120-6-58)

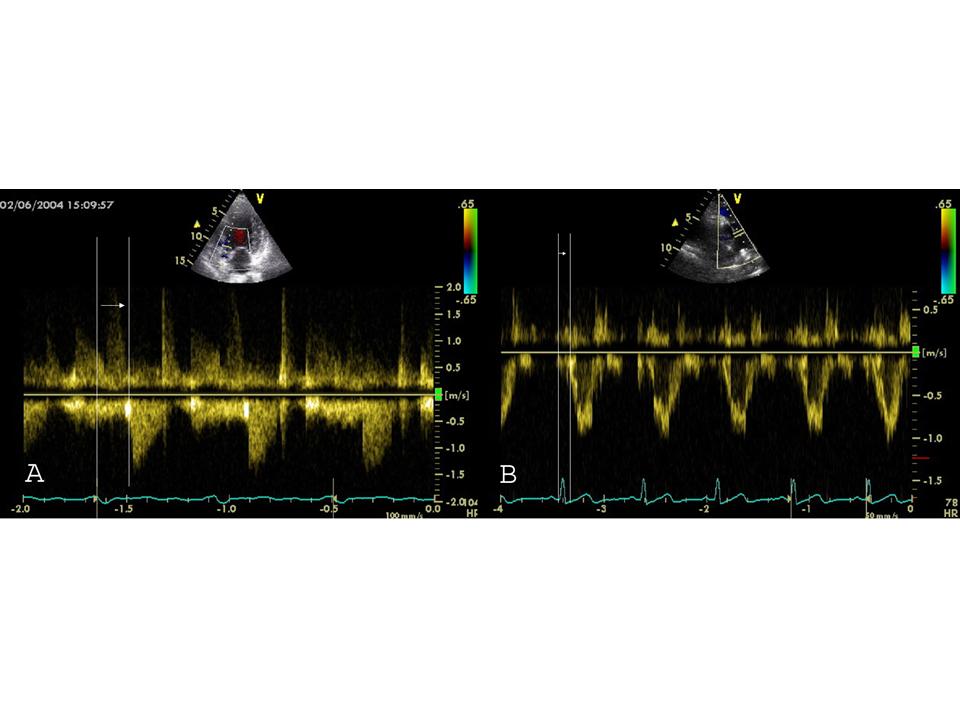

Supplement: Additional File 1 — Measurement of the IMD in the aortic and the pulmonary outflow tracts. [file 1476-7120-6-58-S1.jpeg]

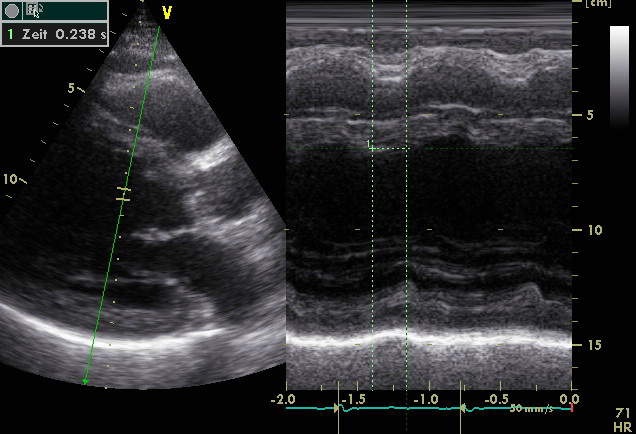

Supplement: Additional File 2 — M-mode in the paarasternal long axis to determine SPWMD. [file 1476-7120-6-58-S2.jpeg]

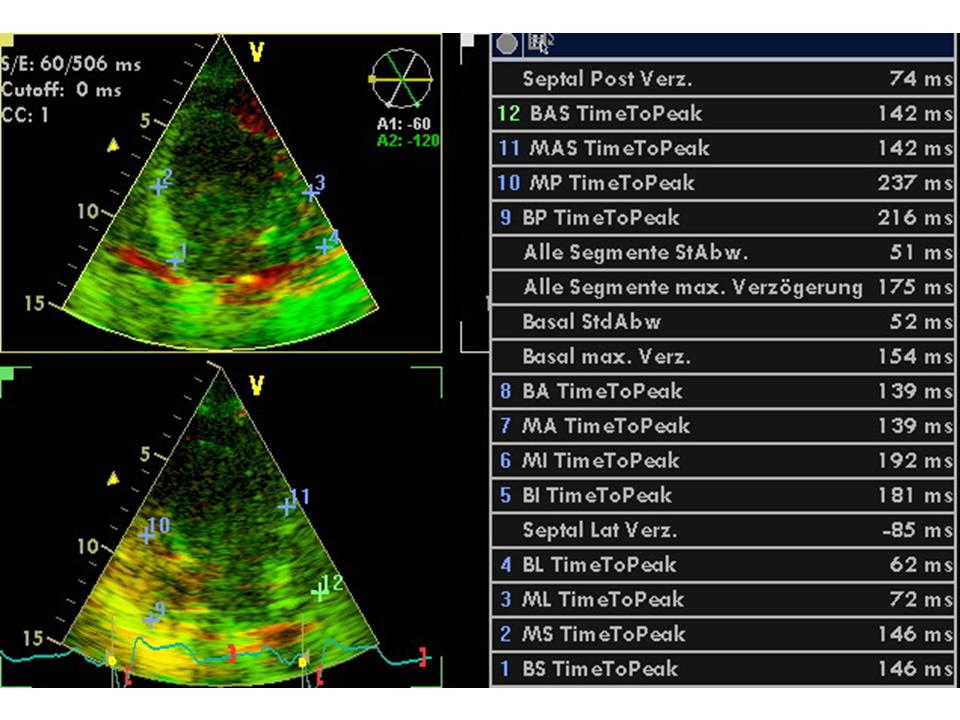

Supplement: Additional File 3 — Assessment of Ts12 and Ts-12-SD by TSI. [file 1476-7120-6-58-S3.jpeg]

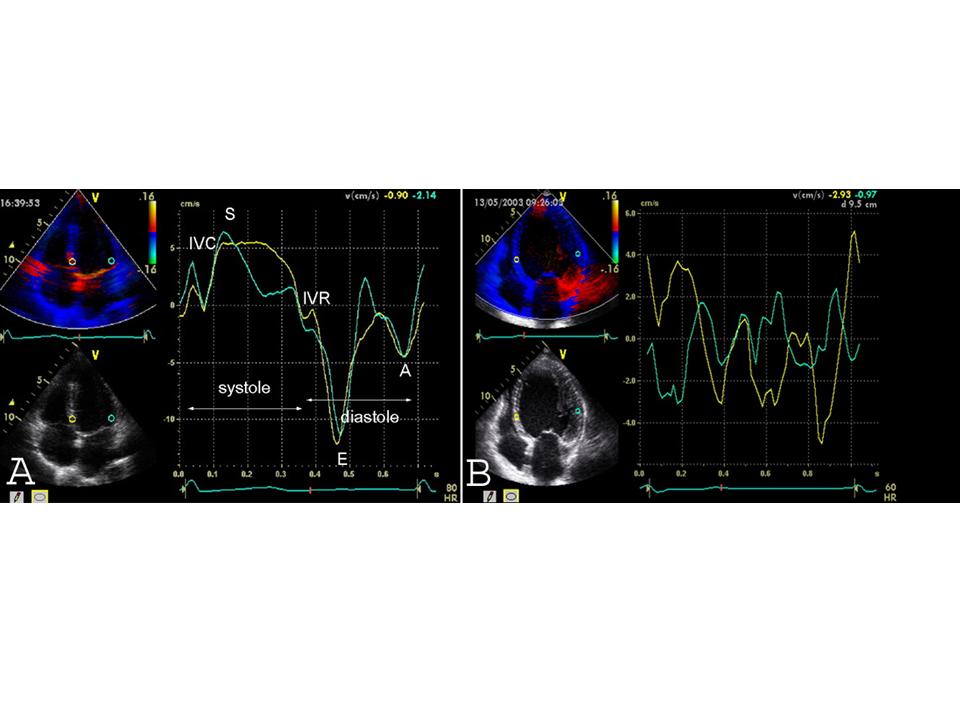

Supplement: Additional File 5 — TDId in a healthy control and the asynchrony of myocardial contraction in the septal and lateral basal segments. [file 1476-7120-6-58-S5.jpeg]

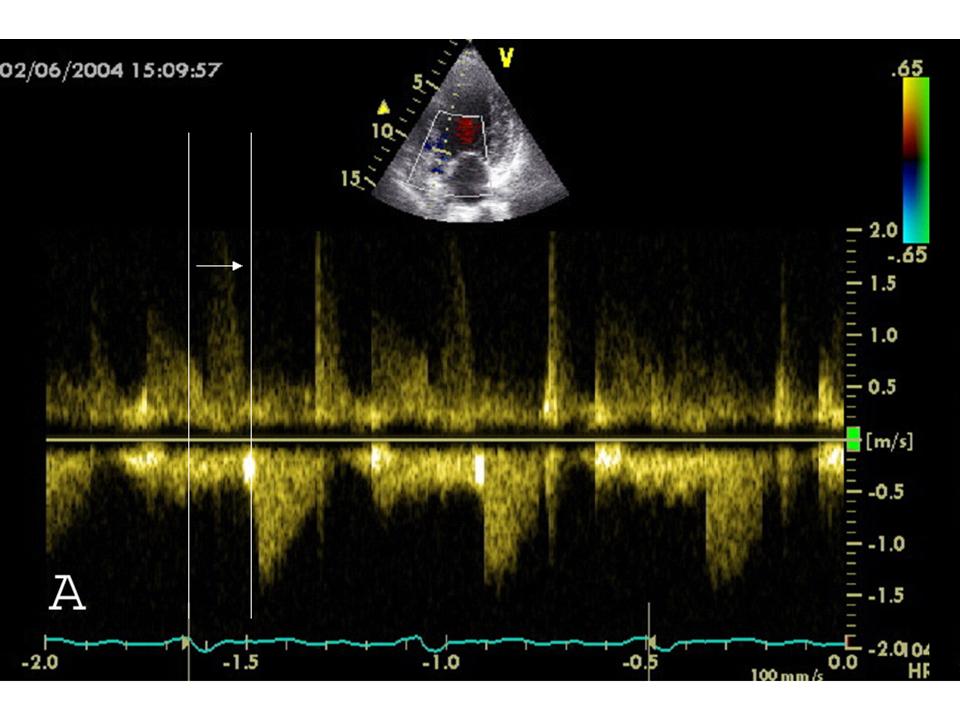

Supplement: Additional File 6 — Measurement of the LVEMD in the aortic outflow tract. [file 1476-7120-6-58-S6.jpeg]
